# IR action spectroscopy of glycosaminoglycan oligosaccharides

**DOI:** 10.1007/s00216-019-02327-7

**Published:** 2019-12-18

**Authors:** Maike Lettow, Márkó Grabarics, Eike Mucha, Daniel A. Thomas, Łukasz Polewski, Joanna Freyse, Jörg Rademann, Gerard Meijer, Gert von Helden, Kevin Pagel

**Affiliations:** 1 Department of Molecular Physics, Fritz Haber Institute of the Max Planck Society, Faradayweg 4-6, 14195 Berlin, Germany; 2 Institute of Chemistry and Biochemistry, Freie Universität Berlin, Takustraße 3, 14195 Berlin, Germany; 3 Institute of Pharmacy, Medicinal Chemistry, Freie Universität Berlin, Königin-Luise-Str. 2+4, 14195 Berlin, Germany

**Keywords:** Glycosaminoglycans, Fondaparinux, Mass spectrometry, Action spectroscopy, Cryogenic infrared spectroscopy

## Abstract

Glycosaminoglycans (GAGs) are a physio- and pharmacologically highly relevant class of complex saccharides, possessing a linear sequence and strongly acidic character. Their repetitive linear core makes them seem structurally simple at first glance, yet differences in sulfation and epimerization lead to an enormous structural diversity with only a few GAGs having been successfully characterized to date. Recent infrared action spectroscopic experiments on sulfated mono- and disaccharide ions show great promise. Here, we assess the potential of two types of gas-phase action spectroscopy approaches in the range from 1000 to 1800 cm^−1^ for the structural analysis of complex GAG oligosaccharides. Synthetic tetra- and pentasaccharides were chosen as model compounds for this benchmark study. Utilizing infrared multiple photon dissociation action spectroscopy at room temperature, diagnostic bands are largely unresolved. In contrast, cryogenic infrared action spectroscopy of ions trapped in helium nanodroplets yields resolved infrared spectra with diagnostic features for monosaccharide composition and sulfation pattern. The analysis of GAGs could therefore significantly benefit from expanding the conventional MS-based toolkit with gas-phase cryogenic IR spectroscopy.

**Electronic supplementary material:**

The online version of this article (10.1007/s00216-019-02327-7) contains supplementary material, which is available to authorized users.

**Figure:**
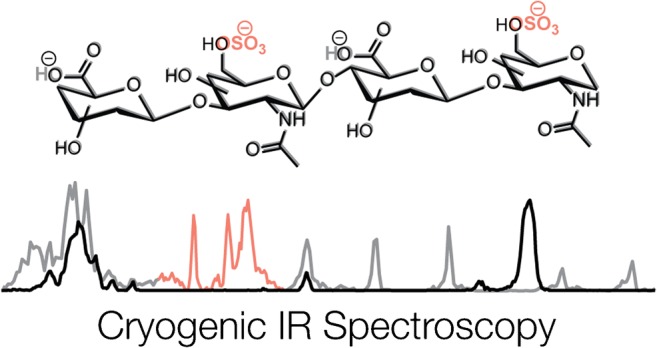
Graphical abstract

## Introduction

Living systems encode information and function in the sequence of biopolymers. Determining the primary structure of nucleic acids and proteins has played a central role in the progress in life sciences, with an arsenal of sensitive and automated sequencing strategies currently available. The high-throughput *de novo* sequencing of glycans, however, is still one of the biggest challenges in bioanalytics [1, 2]. An important group of complex carbohydrates are glycosaminoglycans (GAGs). These are strongly acidic polysaccharides with a linear sequence of repeating disaccharide units [3]. Based on the structure of these disaccharides, five families of GAGs are distinguished (Electronic Supplementary Material (ESM) Figure S1). With the exception of naturally occurring hyaluronan, the backbone is diversely sulfated, giving rise to a multitude of isomeric sequences.

**Fig. 1:**
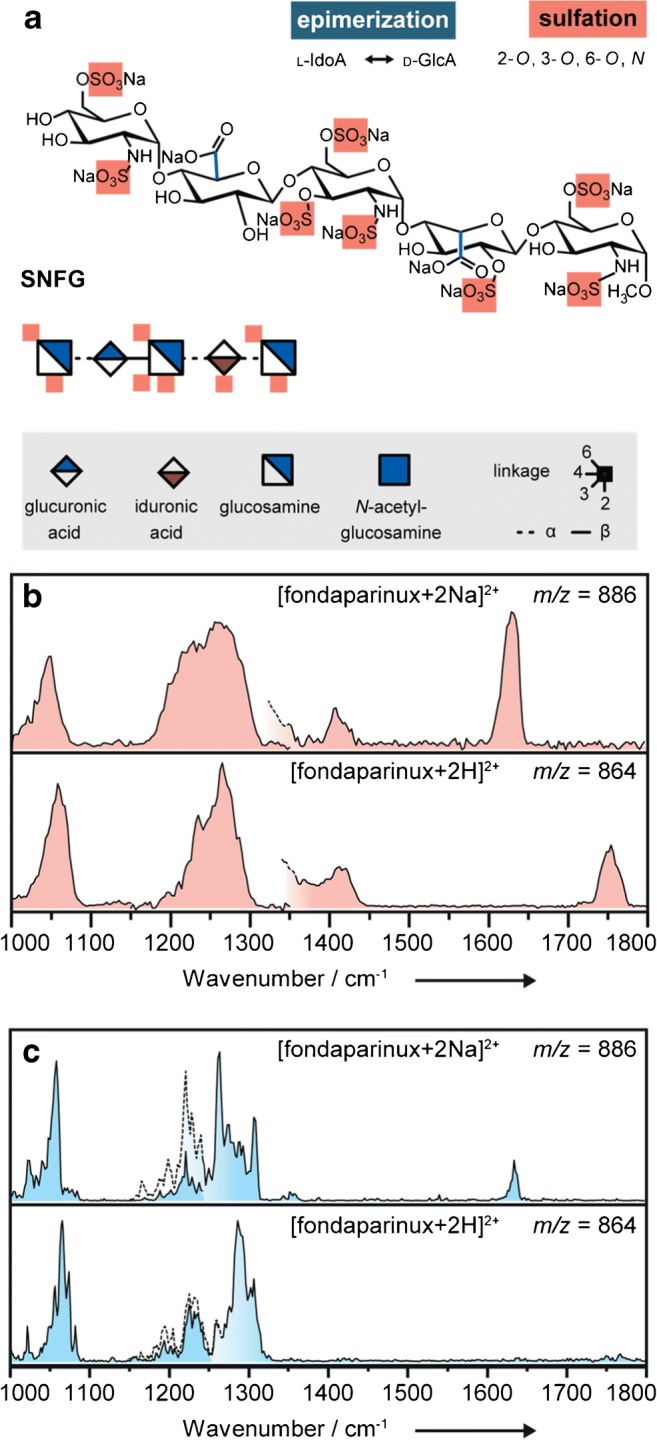
**a** The synthetic anticoagulant fondaparinux (as sodium salt) presented as chemical structure and in the symbol nomenclature for glycans (SNFG, legend given in grey box). Highlighted in red are the positions of the sulfate groups. **b** IRMPD spectroscopy of fondaparinux-sodium salt (1727 Da) investigated as adduct with two additional sodium ions [fondparinux+2Na]^2+^ (upper panel) and as doubly protonated species [fondaparinux+2H]^2+^ (lower panel).**c** Cryogenic IR spectroscopy in helium nanodroplets of the aforementioned ions. Dashed lines indicate an overlap of measurements using different experimental conditions (see ESM)

GAGs are ubiquitous in the extracellular matrix and on cell surfaces [4]. Both as glycoconjugates and in unconjugated form, they mediate various physio- and pathophysiological processes, such as haemostasis, inflammation, tumorigenesis or target-cell recognition in infections [5, 6]. Heparin—arguably the best known and pharmaceutically most relevant natural GAG—is a widely used and potent anticoagulant [7]. Due to its unfavorable pharmacokinetic properties, natural heparin is being increasingly substituted by low molecular weight heparins (LMWHs) and synthetic GAG analogues. The best example of the latter approach is fondaparinux (Arixtra®), a heparin-related fully synthetic pentasaccharide, approved by the EMA and the FDA (Fig. 1a).

Improved quality control in pharmaceutical analysis and more broadly the pursuit to elucidate the glycocode, both urge the development of efficient sequencing methods. However, the analysis of GAGs has proven to be extremely challenging. Despite the relatively simple backbone, they exhibit immense complexity, which arises from two aspects of their structure: size polydispersity and sequence microheterogeneity. As polydisperse systems, their degree of polymerization is not well-defined, making efficient separation methods essential. Sequence microheterogeneity, on the other hand, is the result of uronic acid epimerization and the extent and position of sulfation. Multidimensional workflows combining chromatographic or electrophoretic separations with mass spectrometry (MS) have been traditionally employed to tackle the aforementioned difficulties [8, 9]. Here, the high charge density and the instantaneous, often unintended loss of sulfates during ion manipulation cause additional problems. In recent years, significant progress has been made in using novel electron-based ion activation methods (EDD [10], EID [11], NETD [12]) and ultraviolet photodissociation (UVPD) [13] to improve the capabilities of MS to overcome the unique challenges of GAG analysis. Another promising direction is to employ ion mobility spectrometry (IMS) to separate isomers or isomeric fragments prior to MS analysis [14, 15]. A complementary method to MS-based approaches is Fourier transform infrared (FTIR) spectroscopy, which has been employed for the spectroscopic profiling of intact biological samples [16, 17]. However, despite the aforementioned successful attempts, GAGs are still largely underexplored.

The potential of gas-phase infrared (IR) spectroscopy on mass-selected ions to tackle the complexity of GAGs has been demonstrated using infrared multiple photon dissociation (IRMPD) spectroscopy [18, 19] and messenger-tagging spectroscopy [20]. Generally, spectra measured at low temperatures and without excessive ion heating proved to be better resolved. Here, we employ IRMPD spectroscopy and cryogenic IR spectroscopy in helium nanodroplets in a comparative manner to benchmark the analytical performance of each method. The aforementioned spectroscopic studies focused on disaccharides. Here, we extend the molecular space and report gas-phase IR spectra for synthetic tetra- and pentasaccharides with a different degree of sulfation. Such well-defined structures are well-suited for proof-of-principle analyses, exhibiting every aspect of sequence microheterogeneity, including uronic acid stereochemistry. In addition, the investigated molecules are similar in size to the smallest GAG oligosaccharides that carry biological information [21]. Finally, they provide residue overlap that is essential for sequencing longer GAG chains based on their characteristic fragments.

## Experimental methods

Experimental details on sample preparation, IRMPD spectroscopy and cryogenic IR spectroscopy in helium nanodroplets can be found in the ESM. In brief, the experiments utilized in this work comprise of two types of action spectroscopy. The main difference is the type of *action*, namely dissociation versus ejection from a helium nanodroplet (ESM Figure S2). The former is ideal for ions with a low-energy dissociation channel to prevent spectral broadening resulting from unintended ion heating. It is well-known that highly sulfated glycosaminoglycans, both as protonated and deprotonated ions, are challenging to analyze with mass spectrometry-based techniques because of their fragility [22]. However, this fragility can be highly advantageous for a dissociation-based action spectroscopic method, such as IRMPD spectroscopy. Cryogenic IR spectroscopy in helium nanodroplets, on the other hand, is performed on ions cooled to 0.4 K. As a result, highly resolved spectra are typically obtained, which enable the differentiation of minute structural differences in isomeric glycan ions and could potentially serve as fingerprints for identification [23, 24].

## Results and discussion

First, the commercially available fondaparinux-sodium salt (1727 Da, Fig. 1a) was investigated as doubly protonated species [fondaparinux+2H]^2+^ (*m/z* 864) and as adduct with two additional sodium ions [fondaparinux+2Na]^2+^ (*m/z* 886). Using IRMPD, IR spectra for both species were recorded (Fig. 1b). Activation of both precursor ions leads to the loss of one of the eight possible sulfate groups as neutral SO_3_. The level of activation, i.e., the number of photons exciting the molecule, is tuned such that ideally only one loss channel is populated. Both spectra show three regions of absorptions. Most specific for the investigated ions are the positons of bands above 1600 cm^−1^, a region that is typically attributed to the stretching vibrations of carbonyl and carboxylate functional groups. The [fondaparinux+2H]^2+^ ion exhibits a strong absorption centered at 1755 cm^−1^, which indicates that two protons are located at the carboxyl functional groups, making them neutral. In comparison, the sodiated analogue [fondparinux+2Na]^2+^ shows a strong stretching vibration of the charged carboxylates at 1630 cm^−1^. Between 1200 and 1450 cm^−1^, mainly the antisymmetric SO_3_^−^ stretching modes from multiple sulfate groups are observable [18], with minor contributions of weak C–H bending vibrations. In the lower wavenumber range, combined C–O and C–C stretching vibrations from the glycan core and also the symmetric SO_3_^−^ stretching modes are typically found. Overall, the spectra are rather congested, especially in the lower wavenumber range.

To qualitatively benchmark the gain in spectral quality at low ion temperature, the IR spectra for both species [fondparinux+2Na]^2+^ and [fondaparinux+2H]^2+^ were recorded using cryogenic IR spectroscopy (Fig. 1c). Generally, the position of the absorption bands obtained in IRMPD is reproduced. Yet, the spectral resolution in the cryogenic IR spectra is much higher even in the lower wavenumber range. A limitation is that the intensity of bands in the higher wavenumber range is much lower and as a consequence the carbonyl vibration above 1750 cm^−1^ cannot be sufficiently resolved.

**Fig. 2:**
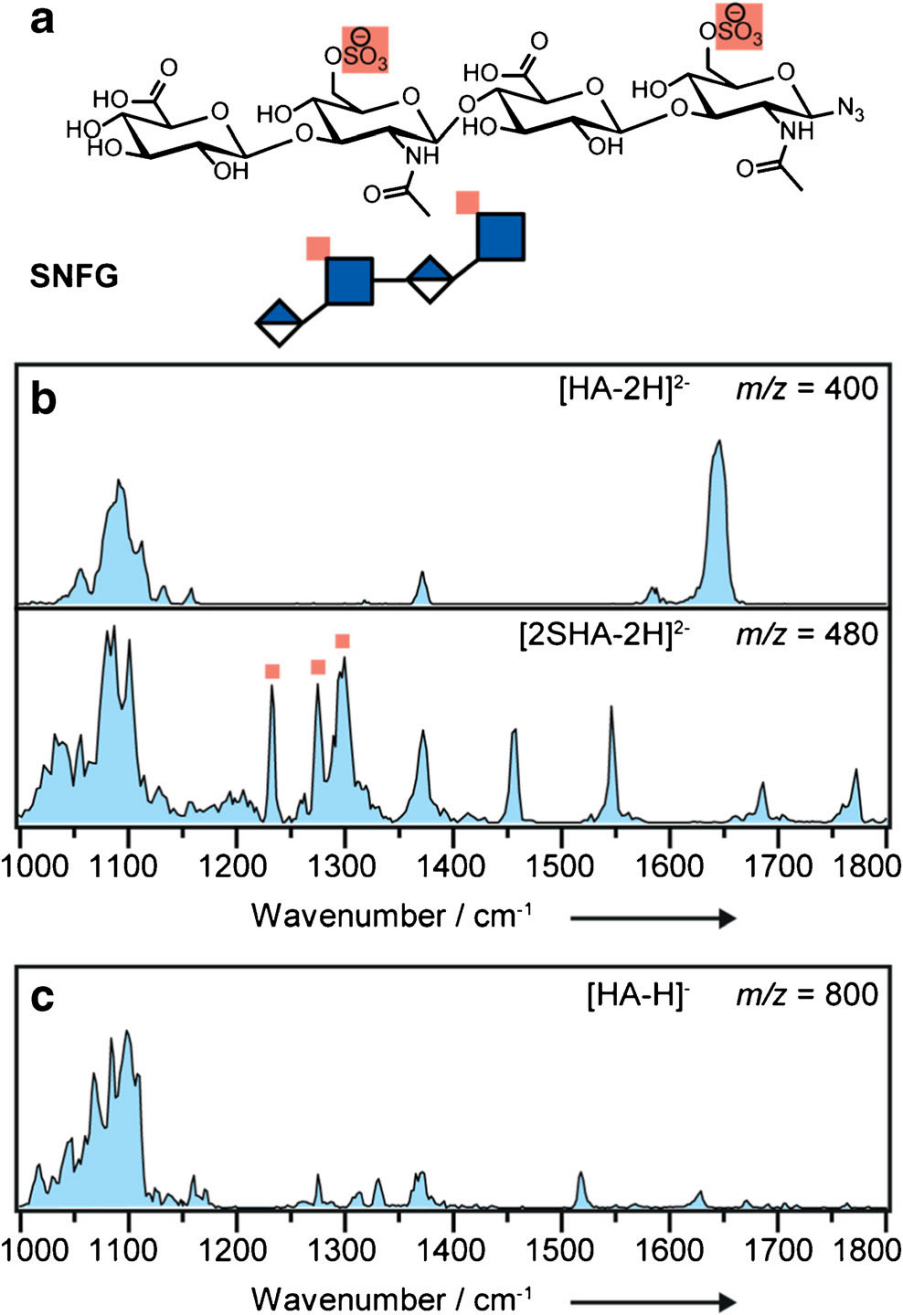
**a** Chemical structure and SNFG representation of the investigated synthetic, sulfated (highlighted in red) hyaluronic acid (SHA) derivative. **b** Cryogenic IR spectroscopy in helium nanodroplets of the non-sufated hyaluronic acid (HA) as [HA-2H]^2−^ (upper panel) and the 2SHA derivative as [2SHA-2H]^2−^ (lower panel). Absorption bands corresponding to sulfate groups are highlighted with red squares. **c** Cryogenic IR spectroscopy in helium nanodroplets of the non-sufated HA as [HA-H]^−^

As cryogenic IR spectroscopy yields spectra of improved resolution, the ability of the method to provide discrete spectral features potentially diagnostic to the sulfation pattern was tested. To do so, a model system of the hyaluronic acid (HA) family was studied. Sulfated hyaluronic acid (SHA) derivatives are in focus of current research to systematically tailor GAG-protein interactions (Fig. 2a) [25]. These newly developed potential drug candidates serve as well-defined standards in this study. With their low degree of sulfation, high stability in a mass spectrometry experiment and acidic nature, negative ion mode is well-suited for these molecules. The IR spectra for the non-sulfated HA tetrasaccharide as [HA-2H]^2−^ (*m/z* 400) and the doubly sulfated 2SHA derivative as [2SHA-2H]^2−^ (*m/z* 480) were recorded using cryogenic IR spectroscopy (Fig. 2b). The strongest absorption in the spectrum of the non-sulfated [HA-2H]^2−^ is the carboxylate antisymmetric stretching vibration at 1645 cm^−1^. The presence of this band confirms the deprotonation of the carboxyl functional group. The amide I band (the C=O stretching vibration of secondary amides) arising from the GlcNAc moiety is most likely overlapped by the strong vibration of the carboxylate anion. The corresponding amide II and III vibrations are assigned to the features observed at 1585 cm^−1^ and 1372 cm^−1^, respectively. Absorptions between 1000 and 1200 cm^−1^ mainly stem from the glycan core.

In order to elucidate the impact of sulfation, the spectrum of the doubly sulfated, but otherwise chemically identical glycan [2SHA-2H]^2−^ was recorded for comparison. As expected from the relative proton affinities of the carboxylate and sulfate moieties, the carboxyl functional groups are neutral, which is confirmed by the C=O stretching vibration at 1770 cm^−1^. The signal at 1685 cm^−1^ is assigned to the amide I vibrations. The amide II and III vibrations can be assigned qualitatively to the bands at 1547 and 1372 cm^−1^. The vibration at 1455 cm^−1^ is in a spectral region in which O–H bending modes in carboxyl groups are typically found. Most importantly, the sulfates show multiple well-resolved bands between 1200 and 1350 cm^−1^ (Fig. 2b, lower panel, highlighted with red squares).

Finding the optimal charge state is crucial to obtaining good spectral quality. With two carboxylate functional groups, the singly deprotonated ion of the tetrasaccharide HA yields a congested spectrum (Fig. 2c). Two chemically almost identical deprotonation sites aid the formation of two *deprotomers*, each with a multitude of individual conformers. In addition, the dense hydrogen bonding network within the molecule can promote charge migration, which further increases the number of potentially observable species [26].

## Conclusion

In summary, we here demonstrate the potential of IR action spectroscopy in the range from 1000 to 1800 cm^−1^ for the structural characterization of highly complex GAG oligosaccharides. Well-resolved IR spectra of oligosaccharides up to pentasaccharides were obtained using cryogenic IR spectroscopy in helium nanodroplets. Signals arising from sulfate groups appear in a spectral range in which typically no other diagnostic vibrations occur. As a result, vibrational patterns with high informational content are highly resolved. The optimal charge state depends on the functional groups present and is crucial for the spectral quality. Besides being sensitive to minute structural differences, cryogenic IR spectroscopy on mass-selected ions has the potential to be implemented in existing MS-based workflows. Current MS-based databases [27] could be extended with IR fingerprints of intact and fragment ions of GAGs. As such, gas-phase IR spectroscopy could serve as a key analytical technique for the characterization of GAG oligosaccharides in the future.

## Electronic supplementary material

ESM 1 (PDF 463kb)
