# Supplementary material for: IR action spectroscopy of glycosaminoglycan oligosaccharides
Source: Anal Bioanal Chem. 2019 Dec 18;412(3):533–7. doi: 10.1007/s00216-019-02327-7 (PMC6992547; doi:10.1007/s00216-019-02327-7)
Supplement: Supplementary file 1 — (PDF 463kb) [file 216_2019_2327_MOESM1_ESM.pdf]

Analytical Bioanalytical Chemistry

Electronic supplementary material

IR action spectroscopy of glycosaminoglycan oligosaccharides

Maike Lettow, Márkó Grabarics, Eike Mucha, Daniel A. Thomas, Lukasz Polewski, Joanna Blaszkiewicz,

Jörg Rademann, Gerard Meijer, Gert von Helden and Kevin Pagel

Experimental methods

Sample preparation

Fondaparinux-sodium salt (Arixtra®) was purchased from Sigma-Aldrich (St Louis, USA). The sulfated oligohyaluronans were synthesized as described previously.[1] All samples were used without further purification. Solvents (HPLC grade) were purchased from Sigma-Aldrich (St Louis, USA). Aqueous glycan stock solutions with a concentration of 1 mM were further diluted prior use with water/methanol (*v*/*v*, 50/50) to yield 50–500 µM analyte solutions.

Infrared multiple photon dissociation spectroscopy

The experimental setup has been described in detail previously[2] and the interested reader is referred to the literature. In brief, glycan ions are generated by a nano-electrospray ion source, transferred into a home-built drift-tube instrument through a capillary source and collected in an ion funnel. The ions are pulsed into the drift tube where they travel through helium buffer gas (~4 mbar) under the influence of a weak, homogenous electric field. In IRMPD experiments, the ions of interest are selected by their drift time and subsequently by their mass-to-charge ratio (*m/z*). In the interaction region of the instrument the glycan ions are irradiated with light in the mid-IR range from 1000 to 1800 cm^{-1} provided by the Fritz Haber Institute's IR free-electron laser (FHI-FEL). Upon irradiation at resonant wavelengths, the energy of the absorbed photons is distributed within the ion through intramolecular vibrational redistribution (IVR) until a barrier for dissociation is reached. The investigated highly fragile GAG ions provide low-energy dissociation channels by loss of neutral SO_3. The parent ion and the respective fragments are detected in a time-of-flight mass analyzer. Action spectra are recorded by plotting dissociation yield as a function of the wavenumber. For each molecule, at least two independent scans are measured and averaged, provided they are reproducible.

The spectrum of the $[\text{fondparinux+2Na}]^{2+}$ ion (Figure 1b) was recorded using lower laser intensity from 1000 to 1350 cm^{-1} and higher intensity from 1350 to 1800 cm^{-1}. Dashed lines in the spectrum indicate an overlap of the measurements. The $[\text{fondaparinux+2H}]^{2+}$ ion (Figure 1b) was recorded using moderate laser intensity from 1000 to 1150 cm^{-1}, lower intensity from 1150 to 1350 cm^{-1} and higher intensity from 1350 to 1800 cm^{-1}.

Cryogenic infrared spectroscopy in helium nanodroplets

The experimental setup utilized in this work has been comprehensively described previously.[3] To give a short description, from an aqueous solution of the aforementioned glycan samples, ions are produced with nano-electrospray and transferred into an in-house built mass spectrometer. The ions of interest are selected by their *m/z* and subsequently stored in a variable-temperature hexapole ion trap. These trapped ions are then captured by traversing superfluid helium nanodroplets in which they are rapidly cooled to 0.37 K. The embedded ions are exposed to tunable and coherent light in the mid-IR range from 1000 to 1800 cm^{-1} provided by the FHI-FEL. After the absorption of multiple photons, the ions are ejected from the droplets, detected in a time-of-flight mass analyzer and analyzed as the integrated ion intensity as a function of the laser's

wavelength. For each molecular ion, at least two independent scans are measured and averaged, provided they are reproducible.

The spectra of [fondparinux+2Na]$^{2+}$ and [fondaparinux+2H]$^{2+}$ (Figure 1c) were recorded using a softer laser focus from 1000 to 1250 cm^{-1} and tighter laser focus from 1250 to 1800 cm^{-1}, to achieve lower and higher photon flux in the interaction region, respectively. Dashed lines in the spectrum indicate an overlap of the measurements. The spectra of [HA-2H]$^{2-}$, [2SHA-2H]$^{2-}$ and [HA-H]$^{-1}$ were recorded using similar laser settings within one spectrum.

Figures

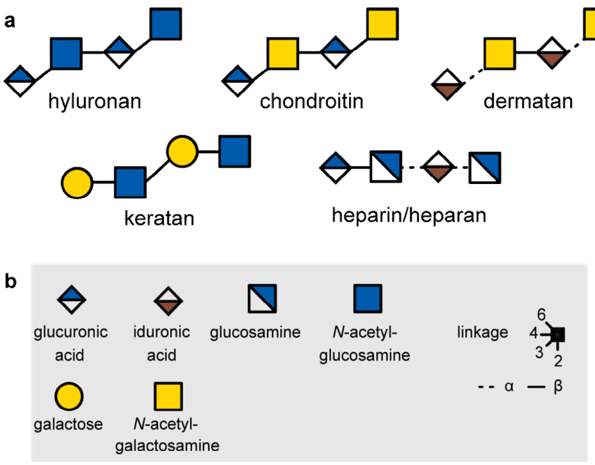

Fig. S1 **a** General structure of the backbone of glycosaminoglycan families. **b** Symbol nomenclature for glycans (SNFG): galactose (Gal), D-glucuronic acid (D-GlcA) or L-iduronic acid (L-IdoA) in the first position; glucosamine (GlcN), *N*-acetylglucosamine (GlcNAc) or *N*-acetylgalactosamine (GalNAc) in the second position of the disaccharide units.

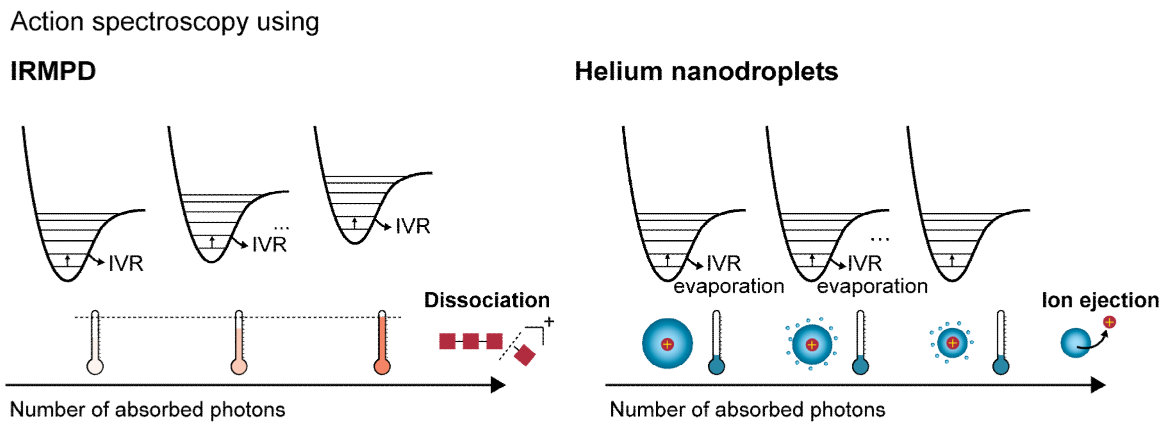

Fig. S2 Comparison of two types of action spectroscopy approaches: infrared multiple photon dissociation spectroscopy (IRMPD, left) and infrared spectroscopy in helium nanodroplets (right).

References

1. Köhling S, Blaszkiewicz J, Ruiz-Gómez G, Fernández-Bachiller MI, Lemmnitzer K, Panitz N et al. Syntheses of defined sulfated oligohyaluronans reveal structural effects, diversity and thermodynamics of GAG–protein binding. Chem Sci. 2019;10(3):866-78.

2. Warnke S, von Helden G, Pagel K. Analyzing the higher order structure of proteins with conformer-selective ultraviolet photodissociation. Proteomics. 2015;15(16):2804-12.

3. Mucha E, Flórez AIG, Marianski M, Thomas DA, Hoffmann W, Struwe WB et al. Glycan Fingerprinting via Cold-Ion Infrared Spectroscopy. Angew Chem Int Ed. 2017;56(37):11248-51.
